# Effect of chitosan and ε-Polylysine composite coating on postharvest quality maintenance and disease resistance of fresh *Tremella fuciformis*

**DOI:** 10.1016/j.fochx.2026.104050

**Published:** 2026-06-03

**Authors:** Yusha Liu, Junzheng Sun, Mengjie Yang, Qiting Li, Shaoxiong Zhou, Chunmei Lai, Yingying Wei, Longxiang Li, Kai Ye, Baosha Tang, Pufu Lai

**Affiliations:** a College of Food Science, Fujian Agriculture and Forestry University, Fuzhou, Fujian 350002, China; b Institute of Food Science and Technology, Fujian Academy of Agricultural Sciences, Fuzhou, Fujian 350003, China; c National R&D Center for Edible Fungi Processing, Fuzhou, Fujian 350003, China; d Key Laboratory of Subtropical Characteristic Fruits, Vegetables and Edible Fungi Processing (Co-Construction by Ministry and Province), Ministry of Agriculture and Rural Affairs, Fuzhou, Fujian 350003, China; e College of Life Science, Fujian Agriculture and Forestry University, Fuzhou, Fujian 350002, China

**Keywords:** *Tremella fuciformis*, Chitosan, Ε-Polylysine, Coating, Disease resistance, Phenylpropanoid metabolism, Transcriptomics

## Abstract

Postharvest quality deterioration limits the commercial value of fresh *Tremella fuciformis*. This study investigated the effects of a chitosan (CTS) and ε-polylysine (ε-PL) composite coating on *T. fuciformis* stored at 25 °C. The treatment significantly inhibited respiration, maintained cell membrane integrity, and reduced the disease index. Biochemically, the coating acted as an exogenous elicitor, triggering early accumulation of hydrogen peroxide (H_2_O_2_) and jasmonic acid (JA). This signaling cascade activated the phenylpropanoid pathway, enhancing key enzyme activities (C4H, 4CL, CAD, LAC) and promoting the synthesis of lignin and antimicrobial phenolic acids. Transcriptomics confirmed the upregulation of genes involved in phenylpropanoid biosynthesis, consistent with enzyme profiles. Thus, the CTS and ε-PL composite coating enhances disease resistance by regulating metabolic flux at the transcriptional level to reinforce physical and chemical barriers, offering an effective preservation strategy.

## 1 Introduction

*Tremella fuciformis* Berk., belonging to the order Tremellales of the phylum Basidiomycota, is an edible and medicinal fungus widely cultivated in China. It is favored by consumers for its unique texture and rich bioactive components, including polysaccharides, dietary fiber, essential amino acids, and trace elements (Chen, 2010; Wu et al., 2019). As the world's largest producer and exporter, China ranks first globally in both the production and sales volume of *T. fuciformis* (Chen et al., 2025). However, fresh *T. fuciformis* poses a significant preservation challenge due to its extremely high water content (>90%), lack of protective epidermal tissues, and vigorous respiratory metabolism (Sun et al., 2024). During postharvest storage, it is highly susceptible to mechanical injury and microbial infection (e.g., molds and yeasts), leading to rapid browning, softening, and decay. Its shelf life at room temperature is typically restricted to 1–3 days (Fan et al., 2017; Wang et al., 2022), which severely limits its market radius and economic value. Therefore, developing efficient, safe, and environmentally friendly preservation technologies is critical for reducing postharvest losses.

Among various preservation strategies, edible coatings have emerged as a research hotspot due to their biodegradability, safety, and cost-effectiveness (Beaulieu et al., 2002; Garcia & Davidov-Pardo, 2021). These coatings not only create a semi-permeable physical barrier to retard water loss and respiration but also serve as carriers for antimicrobial agents (Cavusoglu et al., 2021). Chitosan (CTS), a natural cationic polysaccharide, is renowned for its excellent film-forming properties and broad-spectrum antimicrobial activity, making it widely used in edible fungus preservation (Jiang et al., 2012; Li et al., 2019). ε-Polylysine (ε-PL) is a natural antimicrobial polypeptide produced by *Streptomyces albulus*. Characterized by high water solubility, thermal stability, and safety (FDA-certified GRAS substance), it serves as a potent preservative (Lv et al., 2022; Shu et al., 2021). While single preservatives often show limited efficacy, composite coatings can exert synergistic effects (Wang et al., 2022). Although the combination of chitosan and ε-PL has been applied to crops like winter jujube (Chang & Zhang, 2024), its application on fresh *T. fuciformis* and the underlying mechanisms regarding disease resistance remain underexplored.

Beyond physical barriers, the induced resistance of the host plays a pivotal role in defending against pathogen invasion. Edible fungi possess an innate immune system that can be triggered by external elicitors (such as chitosan). This activation typically involves a cascade of reactions: the burst of reactive oxygen species (ROS), the accumulation of signaling molecules (e.g., H_2_O_2_ and JA), and the subsequent activation of secondary metabolic pathways (Wang et al., 2022a; Yang et al., 2022). The phenylpropanoid pathway serves as the core metabolic route for synthesizing defense-related compounds (Yang et al., 2022). Key enzymes in this pathway, including cinnamate-4-hydroxylase (C4H), 4-coumarate: CoA ligase (4CL), cinnamyl alcohol dehydrogenase (CAD), and laccase (LAC), synergistically catalyze the production of lignin and phenolic acids (Lu et al., 2006; Zhang et al., 2022a). Lignin deposition reinforces the cell wall to form a mechanical barrier, while phenolic acids exhibit direct antimicrobial toxicity (El Modafar & El Boustani, 2001). Thus, elucidating how preservation treatments regulate this metabolic network is essential for understanding the mechanism of extended shelf life.

Despite the widespread use of chitosan and ε-PL, the relationship between their combined application and the active defense metabolism of fresh *T. fuciformis*—particularly the transcriptional regulation of the phenylpropanoid pathway—has rarely been reported. Therefore, this study investigates the effects of the chitosan and ε-PL composite coating on the postharvest quality and disease resistance of fresh *T. fuciformis*. By integrating physiological and biochemical analyses with transcriptomics (RNA-seq), we aim to uncover the molecular mechanisms by which this composite coating regulates gene expression and metabolic flux to promote the synthesis of disease-resistance substances (lignin, jasmonic acid, and phenolic acids). This work provides a theoretical basis for the application of induced resistance strategies in the postharvest preservation of edible fungi.

## 2 Materials and methods

### 2.1 Materials and treatments

Fresh *T. fuciformis* (silver ear mushrooms) were harvested from Gutian County, Ningde City, Fujian Province, China. The mushrooms were harvested at a consistent maturity stage (cultivation time of 35–43 days), characterized by the shrinkage and wrinkling of the cultivation bags. On the day of harvest, the mushrooms were transported to the laboratory. *T. fuciformis* bodies that were disease-free, undamaged, and morphologically intact, with a uniform diameter (12 ± 2 cm) and weight (120 ± 20 g), were selected for the experiment. The selected fresh *T. fuciformis* were randomly divided into two groups and subjected to the following treatments:

(1) Chitosan + ε-PL (CTS + ε-PL) treatment group: The samples were evenly sprayed with a composite preservative solution containing 5 g/L chitosan and 150 mg/L ε-polylysine. The formulation selection was based on our preliminary screening of various CTS (1, 5, and 10 g/L) and ε-PL (50, 100, 150, and 200 mg/L) combinations, as well as their single-component treatments. Because the composite coating exhibited superior preservation efficacy compared to the single treatments, the 5 g/L CTS + 150 mg/L ε-PL ratio was selected as it exhibited the optimal balance between antimicrobial efficacy and film-forming properties. Therefore, to deeply elucidate the molecular mechanisms of this highly effective system, the present study focused specifically on comparing the optimal composite treatment group with the control.
(2) Control group: The samples were sprayed with sterile water using the same method.

After spraying, the samples were naturally air-dried at room temperature until surface moisture evaporated (30 min), and packaged in polyethylene film bags. They were then stored in an environment at (25 ± 1) °C with a relative humidity of 80%. Samples were randomly selected from each treatment daily for observation and determination of relevant indicators.

### 2.2 Determination of post-harvest quality indicators

#### 2.2.1 Sensory analysis

Sensory analysis of fresh *T. fuciformis*, including texture, odor, color, appearance, and overall score, was performed according to the method of Yang et al. (2025). The evaluation was conducted using a 9-point scale, and the overall score was calculated based on the following weights: color (30%), odor (20%), texture (30%), and appearance (20%). The samples were evaluated by a trained panel of 10 members. Formal ethical approval was exempt per national laws and institutional guidelines. This routine evaluation involved safe, food-grade mushrooms treated with CTS and ε-PL with no health risks. Appropriate protocols were utilized to protect the rights and privacy of all participants, with no personal information disclosed, and all provided informed consent. Prior to evaluation, the fresh *T. fuciformis* samples were kept in closed, odorless plastic containers at room temperature. Each treatment was evaluated within 2 h to avoid the development of off-odors.

The specific scoring criteria were as follows:

Color: A score of 7–9 indicated a uniform milky white color; 4–6 indicated uneven color; and < 3 indicated an uneven deep yellow color.

Odor: A score of 7–9 indicated the characteristic aroma of *T. fuciformis*; 4–6 indicated no special aroma; and < 3 indicated a malodor.

Texture (Tactile): A score of 7–9 indicated a soft texture; 4–6 indicated dry and hardened tissue; and < 3 indicated melting due to decay.

Appearance: A score of 7–9 indicated good overall appearance with no damage; 4–6 indicated the presence of some surface defects; and < 3 indicated severe surface damage and poor integrity.

#### 2.2.2 Disease index

Referring to the method of Jiang et al. (2024a), 50 fresh *T. fuciformis* fruit bodies were randomly selected for evaluation. The severity of the disease was categorized into five grades (0–4) based on the proportion of the lesion area (yellowing and rotting) to the total surface area. The grading standards were defined as follows:

Grade 0: No yellowing or rotting lesions.

Grade 1: Lesion area accounts for <1/4 of the total area.

Grade 2: Lesion area is ≥1/4 but <1/2 of the total area.

Grade 3: Lesion area is ≥1/2 but <3/4 of the total area.

Grade 4: Lesion area is ≥3/4 of the total area.

The disease index was calculated using the following formula:

$$\mathrm{Disease Index}=\frac{\sum \left(\mathrm{Disease grade}\times \mathrm{Number of fruiting bodies}\;\mathrm{at}\;\mathrm{this grade}\right)}{\mathrm{Total number of fruiting bodies}\times \mathrm{Highest disease grade}}$$

#### 2.2.3 Respiration rate

To determine the respiration rate, the method described by Xia et al. (2023) was followed. Ten fresh *T. fuciformis* fruit bodies were weighed and placed in a 2 L respiration chamber. The respiration rate was measured using a respiration rate analyzer and expressed as mg/kg·h.

#### 2.2.4 Relative electrical conductivity

Relative electrical conductivity was measured according to the method of Yan et al. (2021). Fresh *T. fuciformis* tissue (5 g) was weighed and immersed in 25 mL of distilled water. After standing at 25 °C for 3 h, the electrical conductivity of the exudate (*R*_*0*_) was measured using a conductivity meter. The mixture was then placed in a boiling water bath for 30 min. After cooling to room temperature, distilled water was added to restore the volume to 25 mL. The solution was mixed well, and the electrical conductivity (*R*_*1*_) was measured. The relative electrical conductivity was calculated using the following formula:

$$\text{Relative Electrical Conductivity}\left(\%\right)=\frac{R_{0}}{R_{1}}\times 100\%$$

### 2.3 Determination of disease resistance-related substances and signaling molecules

#### 2.3.1 Lignin content

Lignin content was determined referring to the method of Wang et al. (2025). Fresh *T. fuciformis* samples were dried to a constant weight. A 5 mg sample was mixed with 1.5 mL of 80% ethanol and incubated in a water bath at 50 °C for 20 min. After cooling, the mixture was centrifuged at 10,000 ×*g* for 10 min at 25 °C, and the supernatant was discarded. The precipitate was mixed with 1 mL of 80% ethanol, incubated in a water bath at 50 °C for 20 min, cooled, and centrifuged again at 10,000 ×*g* for 10 min at 25 °C. The precipitate was dried and dissolved in 750 μL of 25% acetyl bromide in glacial acetic acid. The mixture was incubated in a water bath at 70 °C for 30 min. Subsequently, 300 μL of a mixed solution (2 mol/L NaOH and 7.5 mol/L hydroxylamine hydrochloride) and 450 μL of glacial acetic acid were added and mixed. The mixture was centrifuged at 9000 ×*g* for 10 min at 25 °C. The absorbance of the supernatant was measured at 280 nm. Lignin content was expressed as mg/g dry weight (DW).

#### 2.3.2 Hydrogen peroxide (H_2_O_2_) content

According to the method of Tang et al. (2022), 0.1 g of fresh *T. fuciformis* was homogenized with 1 mL of acetone. The homogenate was centrifuged at 8000 ×*g* for 10 min at 4 °C. The supernatant (0.25 mL) was collected and mixed with 0.025 mL of titanium tetrachloride solution and 0.05 mL of concentrated ammonia. The mixture was centrifuged at 8000 ×*g* for 10 min at 25 °C, and the supernatant was discarded. The precipitate was dissolved in 0.25 mL of sulfuric acid and allowed to stand at room temperature for 5 min. The absorbance was measured at 412 nm. The content of H_2_O_2_ was expressed as μmol/g fresh weight (FW).

#### 2.3.3 Ferulic acid and Caffeic acid

Referring to Wang et al. (2022b), 0.2 g of fresh *T. fuciformis* was weighed and mixed with 1 mL of 80% methanol. The mixture was ultrasonicated in an ice-water bath for 1 h and extracted overnight at 4 °C. After centrifugation at 8000 ×*g* for 10 min at 4 °C, the supernatant was filtered through a syringe filter and analyzed using high-performance liquid chromatography (HPLC).

#### 2.3.4 Jasmonic acid (JA) content

JA content was measured following the method of Jiang et al. (2024b). Fresh *T. fuciformis* (0.5 g) was mixed with 4.5 mL of phosphate-buffered saline (PBS) and centrifuged at 8000 ×*g* for 15 min at 4 °C. The supernatant (10 μL) was mixed with 40 μL of diluent and 100 μL of horseradish peroxidase (HRP)-labeled detection antibody. The mixture was incubated in a thermostat at 37 °C for 1 h. After incubation, tetramethylbenzidine (TMB) substrate was added and incubated at 37 °C for 15 min, followed by the addition of sulfuric acid to stop the reaction. The absorbance was measured at 450 nm. JA content was expressed as ng/g FW.

### 2.4 Assay of Phenylpropanoid pathway-related enzyme activities

#### 2.4.1 Cinnamate-4-hydroxylase (C4H) activity

C4H activity was determined according to Guan et al. (2025). Fresh *T. fuciformis* (0.1 g) was homogenized in 1 mL of 200 mM phosphate buffer (pH 7.5, containing 2 mM β-mercaptoethanol) and centrifuged at 10,000 ×*g* for 10 min at 4 °C. The supernatant served as the enzyme extract. The reaction mixture consisted of 10 μL of enzyme extract and 190 μL of reaction solution (containing 2 mL of 50 mM phosphate buffer, 1 mL of 2 mM trans-cinnamic acid, 100 μL of 0.5 mM NADP-Na_2_, and 100 μL of 0.5 mM G-6-P-Na_2_). The absorbance was measured at 340 nm. The values were recorded immediately (OD_1_) and after 5 min (OD_2_). C4H activity was expressed as nmol/min/g FW.

#### 2.4.2 Cinnamyl alcohol dehydrogenase (CAD) activity

Referring to Meng et al. (2025), 0.1 g of fresh *T. fuciformis* was extracted with 1 mL of Tris-HCl buffer (100 mmol/L, pH 8.8) and centrifuged at 10,000 ×*g* for 10 min at 4 °C. The supernatant was collected. The assay was performed by mixing 10 μL of enzyme extract with 190 μL of reaction solution (consisting of 50 μL of 2 mmol/L NADP, 50 μL of 1.0 mmol/L trans-cinnamic acid, and 50 μL of 100 mmol/L phosphate buffer at pH 8.8). The absorbance was measured at 340 nm immediately (OD_1_) and after 5 min (OD_2_). CAD activity was expressed as nmol/min/g FW.

#### 2.4.3 4-Coumarate: Coenzyme a ligase (4CL) activity

According to Meng et al. (2025), 0.1 g of fresh *T. fuciformis* was homogenized in 1 mL of extraction buffer (50 mM Tris-HCl, pH 8.9, containing 15 mM β-mercaptoethanol, 5 mM EDTA, 5 mM Vc, 10 μM Leupeptin, 1 mM PMSF, 0.15% PVP (*w*/*v*), and 30% glycerol) and centrifuged at 10,000 ×*g* for 10 min at 4 °C. The supernatant (10 μL) was mixed with a reaction solution containing 2.2 mL of 5 mM *p*-coumaric acid, 50 mM ATP, 1 mM CoA-SH, and 15 mM MgSO_4_·7 H_2_O. The mixture was reacted at 40 °C for 30 min. The absorbance was measured at 333 nm.

#### 2.4.4 Laccase activity

Laccase activity was measured referring to Zhang et al. (2022b). Fresh *T. fuciformis* (0.1 g) was mixed with 1 mL of sodium acetate solution (0.05 M) and centrifuged at 10,000 ×*g* for 10 min at 4 °C. The supernatant (45 μL) was mixed with 255 μL of reaction mixture (consisting of 0.7 mL ultrapure water, 0.45 mL sodium acetate buffer at 0.1 M, pH 5.0, and 0.15 mL ABTS). The mixture was incubated in a water bath at 60 °C for 20 min and cooled to room temperature. The absorbance was measured at 420 nm. Laccase activity was expressed as nmol/min/g FW.

### 2.5 Transcriptome sequencing and bioinformatics analysis

#### 2.5.1 RNA extraction, library construction, and sequencing

Total RNA was extracted from *T. fuciformis* powder (0.02 g) using Trizol reagent. Briefly, the sample was homogenized in 1 mL of Trizol and extracted for 30 min. After chloroform phase separation and isopropanol precipitation, the RNA pellet was washed with 75% ethanol and dissolved in RNase-free water. RNA integrity was verified by agarose gel electrophoresis. Following quality control, mRNA was enriched from total RNA using Oligo(dT) magnetic beads and fragmented into short sequences (∼300 bp) using fragmentation buffer. First-strand cDNA was synthesized using random hexamer primers, followed by second-strand cDNA synthesis. The double-stranded cDNA was subjected to end-repair, A-tailing, and adaptor ligation. The ligation products were purified and size-selected, followed by PCR amplification to generate the final cDNA library. The library concentration was quantified using Qubit 4.0 and diluted to 1 ng/μL. Sequencing was performed on the NovaSeq X Plus platform (Illumina) to generate paired-end reads.

#### 2.5.2 Bioinformatics analysis

Raw data (fastq format) were processed using SeqPrep and Sickle software to obtain high-quality clean data. The filtering criteria included: (1) removing reads containing adapters; (2) removing reads with >10% unknown bases (N); and (3) removing low-quality reads (bases with *Q*_*phred*_ ≤ 20 accounting for >50%). Clean reads were de novo assembled using Trinity. The assembly quality was optimized and evaluated using TransRate, CD-HIT, and BUSCO. Gene expression levels were calculated using the FPKM (Fragments Per Kilobase of transcript per Million mapped reads) method, with a threshold of FPKM >0.1 for defining expressed genes. Pearson correlation coefficients were calculated using R software to evaluate biological replicate reliability. Differentially expressed genes (DEGs) between groups were identified based on the criteria of adjusted *P*-value (*P*_*adj*_) < 0.05 and Fold Change >2.

#### 2.5.3 Quantitative real-time PCR (qRT-PCR) validation

For qRT-PCR validation, total RNA was extracted from fresh *T. fuciformis* samples using a Broad-spectrum Plant Total RNA Extraction Kit (Shanghai Huiling Biotechnology Co., Ltd.). RNA concentration was measured, and integrity was checked by electrophoresis on an agarose gel with DNA loading buffer. The qualified RNA was stored at −80 °C. First-strand cDNA was synthesized using the First-strand cDNA Synthesis Mix (R0202-100 T, Lablead Biotechnology Co., Ltd.) following the manufacturer's instructions.

Primers for 5 selected genes were designed using the Primer Quest Tool (IDT) and synthesized by Tsingke Biological Technology. The *Actin* gene was used as the internal reference. The primer sequences are listed in Table 1. qRT-PCR was performed using the 2× Visual Realab Green PCR Fast Mixture (R0202–02, Rambolid Biotechnology Co., Ltd.).

**Table 1:** Primer sequences used in RT-qPCR.

Primer name	Primer sequence
Actin	Forward: GGAGAAGATTTGGCATCACACA
	Reverse: GAAGAGCGAAACCCTCGTAGA
TRINITY_DN3729_c1_g2 (TfCAD)	Forward: AGTCTGTCGGTGTCTCTAAC
	Reverse: CGGTGATGTGGATGTTCTTC
TRINITY_DN726_c0_g1 (TfLAC3)	Forward: TGAGGTTGACGACAGATACA
	Reverse: TGGCACTCGGCTGTATTA
TRINITY_DN29799_c0_g2 (TfLAC5)	Forward: TACCTGACGAGAAGAAGGATAA
	Reverse: TGGAGAGGATGAGCAACA
TRINITY_DN24285_c0_g1 (Tf4CL)	Forward: CCAACTCAAGAACGATTGAGA
	Reverse: GGATCGCCTCGATGACTA
TRINITY_DN8202_c0_g1 (TfC4H)	Forward: CTATGGCCGTCACCAATAC
	Reverse: GGTTCATCGAGCAGTTGAT

### 2.6 Data processing and statistical analysis

All experiments were performed in triplicate. Statistical data analysis, including correlation analysis and the determination of significant differences, was conducted using *jamovi* software (Version 2.6). One-way analysis of variance (ANOVA) followed by Tukey's post-hoc test was used to compare the means. Differences were considered statistically significant at *P* < 0.05.

## 3 Results

### 3.1 Effect of chitosan and ε-Polylysine composite treatment on postharvest quality and physiological attributes of Fresh T. Fuciformis

#### 3.1.1 Sensory analysis and disease index

**Fig. 1:**
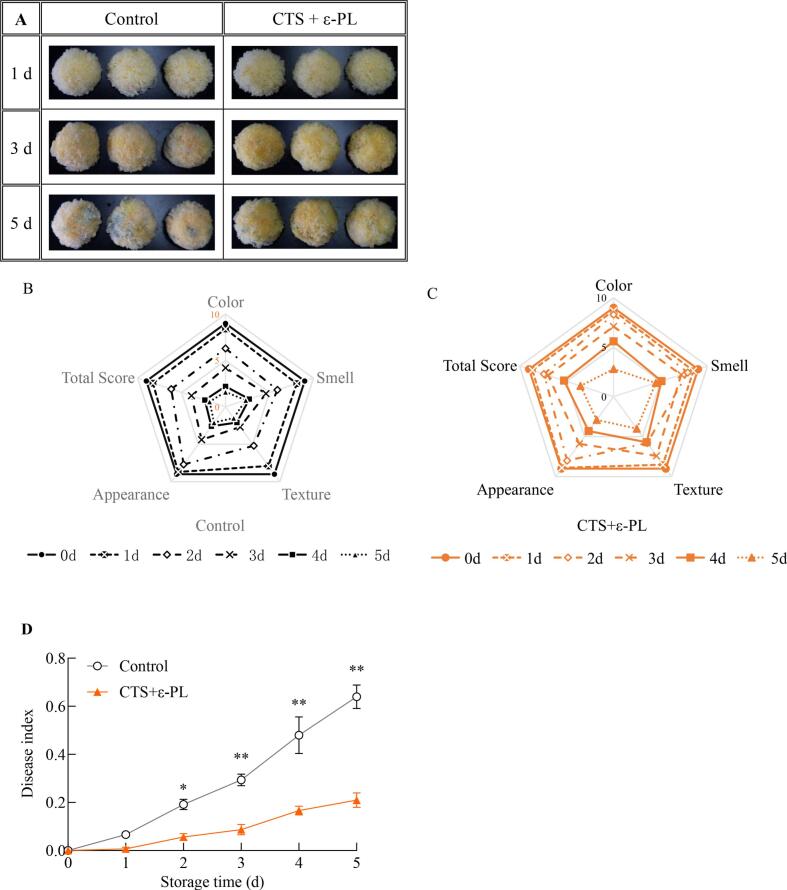
Effects of chitosan (CTS) and ε-polylysine (ε-PL) composite coating on the sensory quality and disease development of fresh *T. fuciformis*. Macroscopic photographs showing the appearance changes of control and CTS + ε-PL treated samples during storage (A); Sensory Analysis of CTS + ε-PL treatment group (B); Sensory Analysis of control group (C). Note: Sensory attributes were evaluated on a 10-point scale, where the center ‘0’ represents decay/inedibility and the outermost axis ‘10’ represents the maximum freshness/quality score. Disease index (D). Asterisks indicate significant differences compared to the control (*P < 0.05, **P < 0.01).

The postharvest quality and resistance of *T. fuciformis* during storage were assessed using macroscopic observation, sensory evaluation, and disease index analysis. As illustrated in the photographs (Fig. 1A), the control group exhibited rapid deterioration; while initially fresh, uneven surface discoloration and yellowing appeared by day 3, followed by severe browning and visible fungal decay by day 5. In contrast, the CTS + ε-PL treatment effectively preserved the characteristic milky white color and structural integrity of the mushrooms, with no visible signs of spoilage during the first 3 days. These visual findings were further quantified using a 10-point sensory evaluation scale (Fig. 1B and C), where a score of 10 represents maximum freshness. The radar charts show that the control group's sensory scores for color, texture, and aroma plummeted after day 2, whereas the treated group maintained high scores throughout the mid-storage period, effectively delaying the onset of senescence by approximately 3 days compared to the control.

These visual observations were corroborated by the disease index analysis (Fig. 1D). While the disease index increased in both groups with prolonged storage, the values in the CTS + ε-PL group were consistently lower than those in the control. A significant difference (*P* < 0.05) was observed as early as day 2, and this difference became highly significant (*P* < 0.01) from day 3 to day 5. Specifically, the control group showed a rapid rise in disease severity, with the index reaching 0.29 by day 3 and 0.64 by day 5. Conversely, the treated group showed no distinct disease symptoms during the first 3 days, and the index reached only 0.21 at the end of the storage period (day 5). These findings demonstrate that the CTS + ε-PL treatment significantly mitigates quality deterioration and exhibits a superior protective effect against pathogen infection.

#### 3.1.2 Respiration rate and relative electrical conductivity

**Fig. 2:**
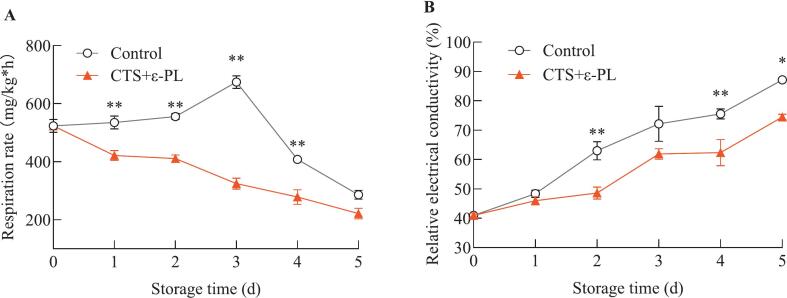
Effects of CTS and ε-PL composite coating on the physiological metabolism of fresh *T. fuciformis*. Respiration rate (A); Relative electrical conductivity (B). Asterisks indicate significant differences compared to the control (*P < 0.05, **P < 0.01).

Respiration intensity is a critical indicator of postharvest metabolic activity. As shown in Fig. 2A, the control group exhibited a typical respiratory climacteric pattern, characterized by a rapid increase peaking on day 3, followed by a sharp decline. In contrast, the CTS + ε-PL treatment effectively suppressed this respiratory surge. The treated group maintained a slow, gradual downward trend throughout the storage period. Consequently, the respiration rate of the treatment group was significantly lower (*P* < 0.01) than that of the control from day 1 to day 4. These results indicate that the composite coating effectively inhibited the vigorous respiratory metabolism of *T. fuciformis*, thereby reducing nutrient consumption and delaying senescence.

Relative electrical conductivity was measured to assess cell membrane permeability and integrity (Fig. 2B). While both groups showed an upward trend over the 5-day storage period, the control group exhibited a rapid increase in conductivity, suggesting severe membrane damage and electrolyte leakage. Conversely, the CTS + ε-PL treatment significantly retarded this increase. The rise in conductivity in the treated group was notably slower, particularly during the first two days. Highly significant differences (*P* < 0.01) between the groups were observed on days 2 and 4. By the end of storage (day 5), the conductivity in the treatment group reached 74.50%, which was significantly lower (*P* < 0.05) than the 87.10% observed in the control group. These findings suggest that the CTS + ε-PL coating effectively maintains cell membrane structural integrity and alleviates oxidative damage during storage.

### 3.2 Induction of signaling molecules: H_2_O_2_ and JA

**Fig. 3:**
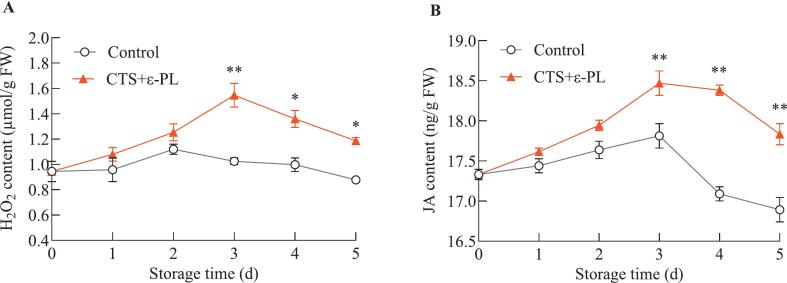
Effects of CTS and ε-PL composite coating on the accumulation of defense signaling molecules. Hydrogen peroxide (H_2_O_2_) content (A); Jasmonic acid (JA) content (B). Asterisks indicate significant differences compared to the control (*P < 0.05, **P < 0.01).

H_2_O_2_ serves as a crucial signaling molecule involved in the oxidative burst, which triggers downstream defense responses. As shown in Fig. 3A, the H_2_O_2_ content in both groups initially increased and subsequently declined. However, the CTS + ε-PL treatment elicited a stronger and more sustained accumulation of H_2_O_2_. Notably, the treatment delayed the appearance of the peak by one day compared to the control (Day 3 vs. Day 2). The peak value in the treatment group reached 1.55 μmol/g, which was approximately 38.39% higher than that of the control (1.12 μmol/g), representing a highly significant difference (*P* < 0.01). The treatment group maintained significantly higher H_2_O_2_ levels in the later stages of storage. These results suggest that the composite coating effectively induces the production of H_2_O_2_, potentially priming the mushroom's defense system.

JA is another key phytohormone regulating immune responses. As depicted in Fig. 3B, JA content followed a trend similar to that of H_2_O_2_, rising initially and then falling. The CTS + ε-PL treatment consistently maintained higher JA levels throughout the storage period. While both groups reached their maximum JA content on day 3, the peak value in the treatment group (18.47 ng/g) was significantly higher (*P* < 0.01) than that in the control group (17.81 ng/g). The difference between the two groups became more pronounced after day 3, remaining highly significant (*P* < 0.01) until the end of storage. These findings indicate that the CTS + ε-PL treatment effectively promotes JA biosynthesis and maintains it at a high level, which may further activate downstream disease resistance pathways.

### 3.3 Activation of Phenylpropanoid metabolism and accumulation of antimicrobial substances

#### 3.3.1 Activities of key enzymes

**Fig. 4:**
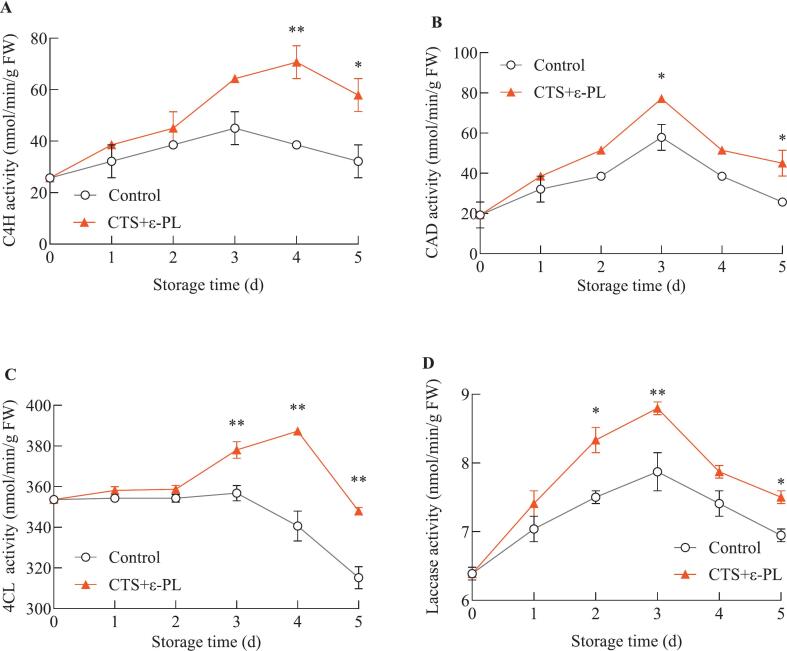
Effects of CTS and ε-PL composite coating on the activities of key enzymes involved in phenylpropanoid metabolism. Cinnamate-4-hydroxylase (C4H) activity (A); Cinnamyl alcohol dehydrogenase (CAD) activity (B); 4-Coumarate: CoA ligase (4CL) activity (C); Laccase (LAC) activity (D). Asterisks indicate significant differences compared to the control (*P < 0.05, **P < 0.01).

Cinnamate-4-hydroxylase (C4H), cinnamyl alcohol dehydrogenase (CAD), 4-coumarate:CoA ligase (4CL), and laccase (LAC) are pivotal enzymes in the phenylpropanoid pathway, governing the synthesis of lignin and phenolic compounds. As illustrated in Fig. 4, the activities of these four enzymes in *T. fuciformis* exhibited a similar pattern: an initial increase followed by a decline during storage. However, the CTS + ε-PL treatment consistently elicited higher enzymatic activities compared to the control.

Specifically, regarding C4H activity (Fig. 4A), the treatment group exhibited a delayed peak on day 4, whereas the control peaked on day 3. The maximum activity in the treated group was 36.36% higher than that of the control (*P*_*adj*_ < 0.01). CAD activity (Fig. 4B) peaked on day 3 for both groups, but the treatment significantly enhanced the peak value by approximately 33.33% (*P*_*adj*_ < 0.05).

A similar trend was observed for 4CL activity (Fig. 4C). The treatment delayed the peak appearance by one day (to day 4) and mitigated the rapid decline observed in the control group. Consequently, a highly significant difference (*P*_*adj*_ < 0.01) was maintained during the late storage period (days 4–5). Laccase activity (Fig. 4D) in the treated group increased rapidly, peaking on day 3 at a level 1.12 times that of the control (*P*_*adj*_ < 0.01). Collectively, these results demonstrate that the CTS + ε-PL composite coating effectively activates and sustains the key enzymatic systems involved in disease resistance metabolism.

#### 3.3.2 Accumulation of lignin and phenolic acids

**Fig. 5:**
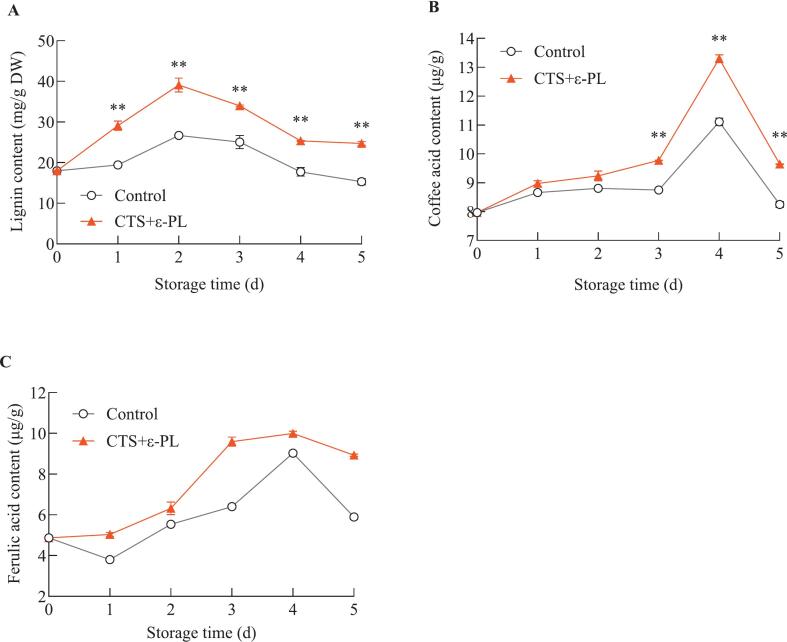
Effects of CTS and ε-PL composite coating on the content of disease resistance-related substances. Lignin content (A); Caffeic acid content (B); Ferulic acid content (C). Asterisks indicate significant differences compared to the control (*P < 0.05, **P < 0.01).

Lignin is a vital component of the cell wall that forms a physical barrier against pathogen invasion. As shown in Fig. 5A, the lignin content in both groups increased initially, peaking on day 2, and then declined. However, the CTS + ε-PL treatment significantly boosted lignin accumulation throughout the storage period (*P*_*adj*_ < 0.01). Specifically, the peak lignin content in the treatment group reached 39.08 mg/g, which was approximately 46.48% higher than that of the control group (26.68 mg/g). This robust accumulation suggests that the composite coating effectively promoted cell wall lignification.

Phenolic acids, such as caffeic acid and ferulic acid, act as precursors for lignin synthesis and possess direct antimicrobial properties. Their changes are illustrated in Fig. 5B and C.

For caffeic acid (Fig. 5B), the treatment group exhibited a rapid increase, peaking on day 4 (13.15 μg/g), whereas the control group peaked earlier on day 3 with a lower value (10.29 μg/g). With the exception of day 3, the caffeic acid content in the treated mushrooms was significantly higher than in the control (*P*_*adj*_ < 0.01).

A similar trend was observed for ferulic acid (Fig. 5C). The content increased continuously until day 4 in the treatment group. Highly significant differences (*P*_*adj*_ < 0.01) between the treatment and control groups were maintained from day 2 to day 4.

Collectively, these results demonstrate that the CTS + ε-PL treatment effectively induces the accumulation of key phenolic acids and lignin, thereby establishing a dual chemical and physical defense system.

### 3.4 Transcriptomic analysis of disease resistance mechanisms

To evaluate the overall reliability and temporal dynamics of the transcriptomic response, global visualization analyses were performed. The principal component analysis (PCA) demonstrated high reproducibility among biological replicates, with samples from different groups clearly separated (Fig. S2). Furthermore, a Venn diagram visualized the overlapping and unique DEGs between day 3 and day 5, highlighting the substantial expansion and temporal shifts of gene expression reprogramming as storage progressed (Fig. S3).

#### 3.4.1 GO and KEGG pathway enrichment analysis

**Fig. 6:**
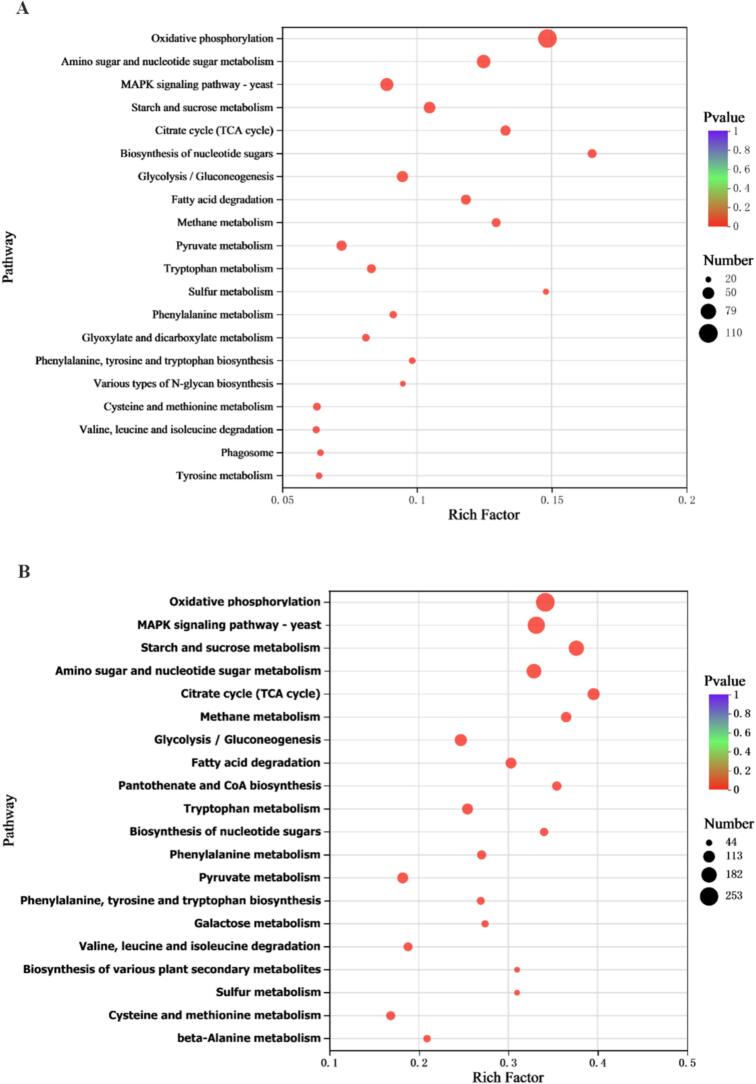
KEGG pathway enrichment analysis of differentially expressed genes (DEGs). Top 20 enriched pathways for DEGs identified on Day 3 (A) and Day 5 (B) of storage. The size of the bubble represents the number of genes, and the color gradient represents the Q-value significance.

To further categorize the broad functions of the DEGs, GO annotation analysis was performed (Fig. S4). The DEGs on both day 3 and day 5 were predominantly classified into major biological categories such as ‘cellular process’, ‘metabolic process’, ‘catalytic activity’, and ‘binding’. Notably, a considerable number of DEGs were also assigned to ‘response to stimulus’ and ‘cellular component organization or biogenesis’. These broad functional classifications provide a foundational macro-perspective that supports the mushroom's physiological responses—such as defense signaling and structural reinforcement—induced by the composite coating. Furthermore, to explore the biological functions of the DEGs induced by the composite coating, KEGG pathway enrichment analysis was performed. As shown in Fig. 6, the DEGs identified on day 3 and day 5 were significantly enriched in metabolic pathways closely related to disease resistance and energy metabolism. Specifically, on day 3 (Fig. 6A), pathways such as oxidative phosphorylation, MAPK signaling pathway–yeast, amino sugar and nucleotide sugar metabolism, and phenylalanine metabolism were significantly enriched. By day 5 (Fig. 6B), the enrichment pattern was sustained, with phenylpropanoid biosynthesis, starch and sucrose metabolism, and phenylalanine, tyrosine, and tryptophan biosynthesis becoming prominent. These results suggest that the CTS + ε-PL treatment modulates the postharvest physiology of *T. fuciformis* by activating signal transduction (MAPK), energy supply (oxidative phosphorylation), and secondary metabolism (phenylpropanoid pathway), which likely contributes to the delayed disease occurrence.

#### 3.4.2 Molecular regulation of Phenylpropanoid pathway and qRT-PCR validation

Based on the physiological data and KEGG enrichment results, we focused on the phenylpropanoid biosynthesis pathway, which is critical for lignin and phenolic acid synthesis.

**Fig. 7:**
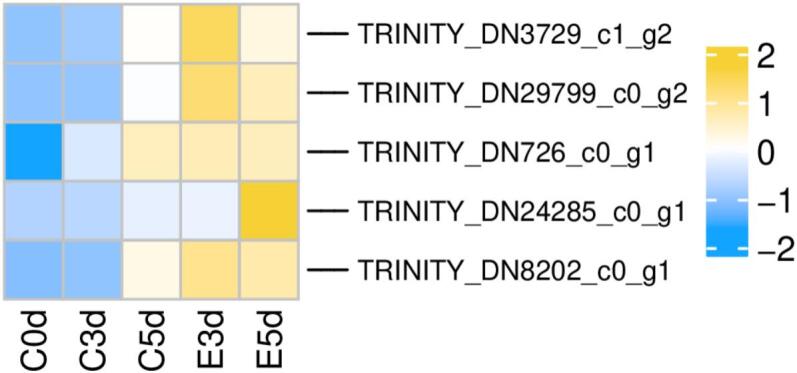
Heatmap visualization of the expression profiles of key genes related to phenylpropanoid biosynthesis. The rows represent individual genes, and the columns represent different samples (Control vs. Treatment at days 0, 3, and 5). The color scale represents the relative expression level, with yellow indicating high expression (upregulation) and blue indicating low expression (downregulation). (For interpretation of the references to color in this figure legend, the reader is referred to the web version of this article.)

Heatmap analysis (Fig. 7) visualized the expression patterns of DEGs encoding key enzymes in this pathway. As shown in the heatmap, genes in the CTS + ε-PL treatment group exhibited distinct upregulation trends compared to the control during storage. Specifically, transcripts annotated as cinnamyl alcohol dehydrogenase (*TfCAD*, TRINITY_DN3729_c1_g2), laccase (*TfLAC5*, TRINITY_DN29799_c0_g2; *TfLAC3*, TRINITY_DN726_c0_g1), 4-coumarate:CoA ligase (*Tf4CL*, TRINITY_DN24285_c0_g1), and cinnamate-4-hydroxylase (*TfC4H*, TRINITY_DN8202_c0_g1) showed significant high-expression profiles in the treated samples.

**Fig. 8:**
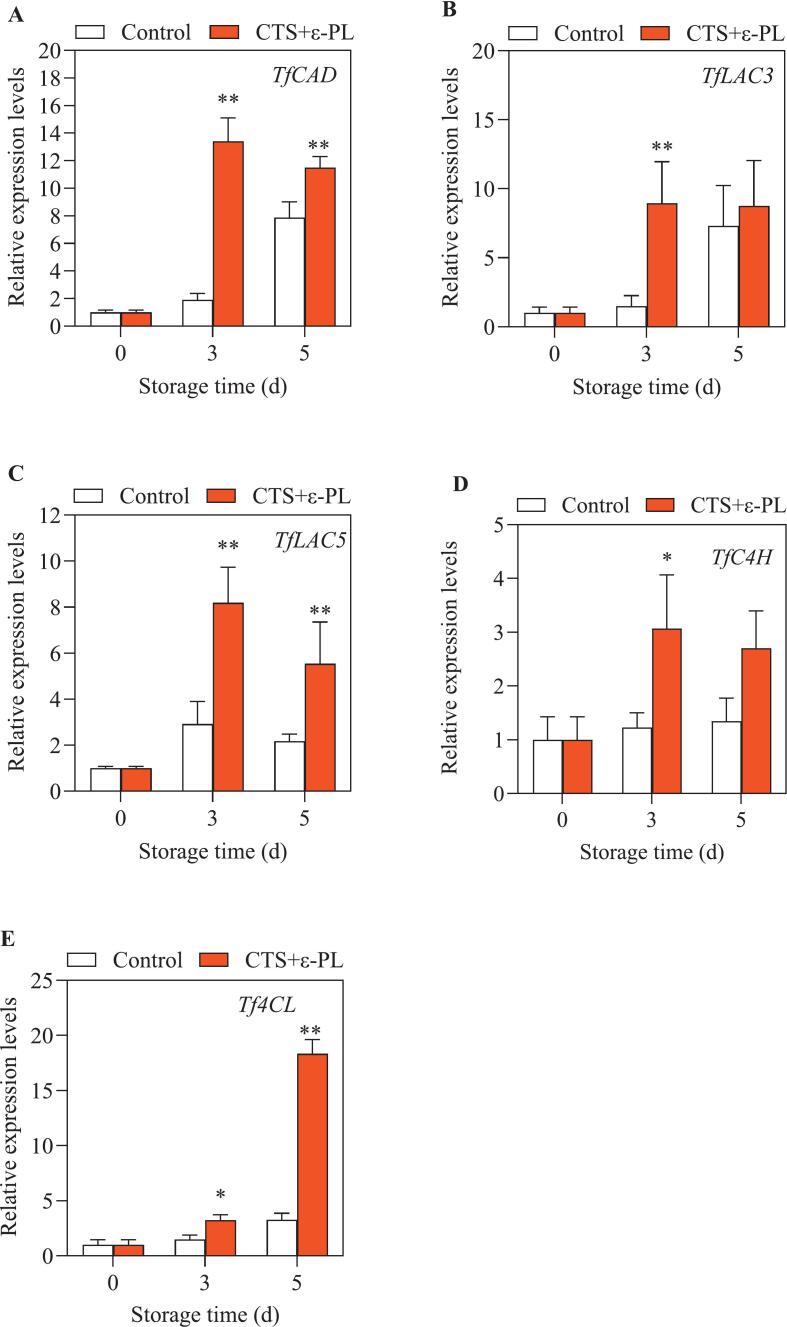
Validation of RNA-seq data by qRT-PCR. Relative expression levels of phenylpropanoid pathway-related genes: *TfCAD* (A), *TfLAC3* (B), *TfLAC5* (C), *TfC4H* (D), and *Tf4CL* (E), in fresh *T. fuciformis* treated with CTS + ε-PL compared to the control. Asterisks indicate significant differences (*P_*adj*_ < 0.05, **P_*adj*_ < 0.01).

To validate the RNA-seq profiles, the relative expression levels of these key genes were quantified using qRT-PCR (Fig. 8). The results showed high consistency with the transcriptomic data:

*TfCAD* (Fig. 8A) and *TfLAC5* (Fig. 8C): Their expression was significantly upregulated (*P*_*adj*_ < 0.01) by the treatment on days 3 and 5. Notably, on day 3, the expression levels in the treatment group were 7.06-fold and 2.81-fold higher than those in the control, respectively.

*TfLAC3* (Fig. 8B) and *TfC4H* (Fig. 8D): These genes also peaked on day 3, showing 7.58-fold and 3.07-fold increases, respectively, compared to the control (*P*_*adj*_ < 0.05).

*Tf4CL* (Fig. 8E): The treatment induced a sustained upregulation of *Tf4CL*, which was 2.17-fold and 5.61-fold higher than the control on days 3 and 5, respectively (*P*_*adj*_ < 0.05).

These findings confirm that the CTS + ε-PL composite coating activates the phenylpropanoid pathway at the transcriptional level. By upregulating the expression of *C4H*, *4CL*, *CAD*, and *LAC*, the treatment drives the metabolic flux toward the synthesis of lignin and antimicrobial compounds, thereby enhancing the intrinsic disease resistance of fresh *T. fuciformis*.

## 4 Discussion

### 4.1 Effects of composite coating on physiological homeostasis and quality maintenance

Respiration is the central pacemaker of postharvest metabolic activity in fresh *T. fuciformis*. Its intensity directly dictates the rate of nutrient consumption and the progression of senescence. In this study, the control group exhibited a typical respiratory climacteric on day 3, a high-intensity metabolic state often accompanied by excessive accumulation of reactive oxygen species (ROS) that accelerates tissue senescence (Zhang et al., 2019). In contrast, the chitosan and ε-polylysine composite coating significantly suppressed the peak respiration rate and delayed the onset of the respiratory climacteric. This effect is primarily attributed to the semi-permeable barrier formed by the coating matrix on the mushroom surface. This physical barrier modifies the gas microenvironment, inducing the tissue to enter a state of relative metabolic quiescence, thereby mitigating physiological deterioration driven by vigorous metabolism.

The integrity of the cell membrane system serves as the primary physical line of defense against pathogen infection. As postharvest senescence progresses, lipid peroxidation disrupts membrane structure, leading to increased cell membrane permeability (manifested as a rise in relative electrical conductivity) and the leakage of intracellular electrolytes. This process not only compromises cellular compartmentalization but also provides nutrient substrates that facilitate pathogen proliferation (Lin et al., 2022). Our results show that the relative electrical conductivity in the treated group was consistently maintained at a lower level. This suggests that by inhibiting respiratory metabolism and oxidative stress, the composite coating effectively preserved membrane integrity, thereby limiting the initial colonization and infection by pathogens. Consequently, the reduced disease index observed in the treatment group is a direct outcome of this enhanced physiological homeostasis combined with the physical shielding effect of the coating.

Furthermore, the composite coating effectively preserved essential nutrients, including polysaccharides, Vitamin C and dietary fiber (Fig. S1), which is consistent with the inhibited respiration rate and maintained physiological homeostasis.

### 4.2 Elicitation of early defense signals: ROS and JA

Beyond its physical barrier properties, chitosan acts as a potent exogenous elicitor that can induce active defense responses in fungi (Guo et al., 2022). The recognition of such elicitors typically triggers a specific signaling cascade, initiating with an oxidative burst and the accumulation of phytohormones.

In this study, the composite treatment induced a rapid and significant accumulation of H_2_O_2_ and JA during the early stage of storage. H_2_O_2_ plays a dual role in defense: it acts as a secondary messenger to activate downstream defense genes and participates directly in cell wall reinforcement via cross-linking (Jongsri et al., 2017). Concurrently, JA serves as a central signaling molecule in coordinating defense responses, particularly against necrotrophic pathogens (Li et al., 2022). The early burst of these signaling molecules suggests a rapid defense initiation. Crucially, our transcriptomic data further elucidates the upstream regulation of this process, as the “MAPK signaling pathway-yeast” was significantly enriched at the same time point (Fig. 6A). As an evolutionarily conserved signal transduction module, the MAPK cascade often functions upstream of the oxidative burst and hormonal signaling in fungal immunity. Therefore, it is highly probable that the CTS + ε-PL coating serves as a molecular elicitor that first triggers the MAPK cascade, which subsequently orchestrates the rapid accumulation of H_2_O_2_ and JA to amplify the defense signal. This early signaling preparation effectively primes the immune system of *T. fuciformis*, shifting it into a defensive alert state prior to severe pathogen infection, and ultimately driving the activation of the downstream phenylpropanoid metabolic defenses.

### 4.3 Coordination of Phenylpropanoid metabolism: Integrating enzyme activities and transcriptional regulation

Driven by the upstream signals (H_2_O_2_ and JA), the phenylpropanoid metabolic pathway—the core battlefield for antimicrobial substance synthesis (Yang et al., 2022)—was significantly activated. The integration of physiological data with transcriptomic analysis reveals a coherent regulatory mechanism governing this activation.

Transcriptomic analysis indicated that the composite treatment induced a comprehensive transcriptional reprogramming, characterized by the significant enrichment of DEGs in the Phenylpropanoid biosynthesis pathway. Specifically, genes encoding key enzymes, including cinnamate-4-hydroxylase (*C4H*), 4-coumarate: CoA ligase (*4CL*), cinnamyl alcohol dehydrogenase (*CAD*), and laccase (*LAC*), were significantly upregulated. This molecular-level regulation directly explains the observed enhancement in enzyme activities. C4H converts cinnamic acid to *p*-coumaric acid, providing the precursor skeleton. Subsequently, 4CL and CAD play pivotal roles in the activation and reduction of hydroxycinnamic acids into lignin monomers, while laccase facilitates the final polymerization of these monomers into lignin (Yu et al., 2022). From a mechanistic perspective, this coordinated transcriptional activation is initiated by the recognition of CTS as an exogenous elicitor by potential receptors on the *T. fuciformis* cell membrane. This elicitor-receptor interaction likely triggers a series of downstream signaling events, including the activation of the MAPK cascade and the production of secondary messengers like H_2_O_2_ and JA. These signals subsequently activate specific transcription factors, which bind to the cis-acting elements in the promoters of phenylpropanoid-related genes (*TfC4H*, *Tf4CL*, *TfCAD*, and *TfLAC*) to initiate their transcription. This regulatory flow explains how the physical application of a CTS-based coating is translated into a robust, genetically-driven biochemical defense response.

The synchronized upregulation of these genes and the subsequent elevation of enzyme activities effectively redirected the metabolic flux toward the synthesis of defensive metabolites. As a result, the treatment group exhibited a higher accumulation of phenolic acids (caffeic acid and ferulic acid) and lignin. Phenolic acids possess direct antimicrobial activity, inhibiting pathogen growth, while lignin deposition reinforces the cell wall, creating a mechanical barrier resistant to enzymatic hydrolysis by pathogens (Jongsri et al., 2017).

In summary, the synergistic effect of the CTS + ε-PL composite coating stems from its ability to construct a “dual barrier” system. Physically, CTS provides a stable structural matrix that modifies the microenvironment to maintain physiological homeostasis, while ε-PL provides broad-spectrum antimicrobial activity by directly inhibiting pathogen proliferation on the mushroom surface. Biologically, the coating acts as a sustained-release carrier and an exogenous elicitor, where CTS triggers the H_2_O_2_ and JA signaling cascade. This activates a “Gene Expression – Enzyme Activation – Metabolite Accumulation” cascade within the phenylpropanoid pathway, leading to the deposition of lignin and the accumulation of phenolic acids to reinforce the endogenous cell wall structure. This “External Physical Protection + Internal Biological Induction” coordination represents a highly efficient strategy for fortifying the intrinsic disease resistance of fresh *T. fuciformis*.

## 5 Conclusion

In conclusion, this study demonstrates that the chitosan and ε-polylysine composite coating serves as an effective preservation strategy to delay senescence and enhance the disease resistance of fresh *T. fuciformis*. Physiologically, the coating forms a semi-permeable barrier that suppresses the respiratory climacteric and mitigates oxidative stress, thereby maintaining cell membrane integrity and inhibiting pathogen colonization. At the molecular and biochemical levels, the composite coating acts as an exogenous elicitor. It triggers an early defense response characterized by the accumulation of signaling molecules, specifically H_2_O_2_ and JA. This signaling cascade induces transcriptional reprogramming, leading to the significant upregulation of genes encoding key phenylpropanoid pathway enzymes (C4H, 4CL, CAD, and LAC). The subsequent increase in enzymatic activities drives the metabolic flux toward the synthesis of antimicrobial phenolic acids and the deposition of lignin, which reinforces the cell wall. Collectively, these findings elucidate that the CTS and ε-PL composite treatment improves postharvest quality through a synergistic mechanism combining physical protection with the active induction of the intrinsic “Signaling–Transcription–Metabolism” defense network.

Beyond the scientific insights, the CTS + ε-PL composite coating holds significant promise for industrial application. Both components are non-toxic, eco-friendly, and cost-effective, meeting the increasing consumer demand for “clean label” food preservatives. The application process—whether through automated spraying or brief dipping—is highly compatible with current fresh mushroom supply chains and cold chain logistics. By maintaining sensory quality and reducing microbial spoilage, this technology provides a sustainable and commercially viable solution to minimize postharvest losses and enhance the market value of fresh *T. fuciformis*.

## CRediT authorship contribution statement

**Yusha Liu:** Writing – review & editing, Writing – original draft, Visualization, Methodology, Data curation, Conceptualization. **Junzheng Sun:** Writing – review & editing, Writing – original draft, Visualization, Methodology, Data curation, Conceptualization. **Mengjie Yang:** Writing – review & editing, Writing – original draft, Data curation, Conceptualization. **Qiting Li:** Writing – review & editing, Methodology. **Shaoxiong Zhou:** Writing – original draft, Methodology. **Chunmei Lai:** Writing – original draft, Data curation. **Yingying Wei:** Writing – review & editing, Methodology. **Longxiang Li:** Writing – review & editing, Methodology. **Kai Ye:** Writing – review & editing, Data curation. **Baosha Tang:** Writing – review & editing, Conceptualization. **Pufu Lai:** Writing – review & editing, Methodology, Data curation, Conceptualization.

## Declaration of competing interest

The authors declare the following financial interests/personal relationships which may be considered as potential competing interests: Yusha Liu, Junzheng Sun, Mengjie Yang, Qiting Li, Shaoxiong Zhou, Chunmei Lai, Yingying Wei, Longxiang Li, Kai Ye, Baosha Tang, Pufu Lai reports financial support and equipment, drugs, or supplies were provided by The National Key Research and Development Program of China (2024YFD2100800). Yusha Liu, Junzheng Sun, Mengjie Yang, Qiting Li, Shaoxiong Zhou, Chunmei Lai, Yingying Wei, Longxiang Li, Kai Ye, Baosha Tang, Pufu Lai reports financial support and equipment, drugs, or supplies were provided by The Fujian Provincial People s Government China Academy of Agricultural Sciences High-quality Development of Agriculture Beyond the 5511 Collaborative Innovation Engineering Project (XTCXGC2021014). Yusha Liu, Junzheng Sun, Mengjie Yang, Qiting Li, Shaoxiong Zhou, Chunmei Lai, Yingying Wei, Longxiang Li, Kai Ye, Baosha Tang, Pufu Lai reports financial support and equipment, drugs, or supplies were provided by The Fujian Province Modern Edible Fungus Industry Technology System Construction Project Minnongkejiao (2024) - grant number 20. Yusha Liu, Junzheng Sun, Mengjie Yang, Qiting Li, Shaoxiong Zhou, Chunmei Lai, Yingying Wei, Longxiang Li, Kai Ye, Baosha Tang, Pufu Lai reports administrative support was provided by The Natural Science Foundation of Fujian Province (2023 J01377). If there are other authors, they declare that they have no known competing financial interests or personal relationships that could have appeared to influence the work reported in this paper.

## Data Availability

Data will be made available on request.
