# Supplementary material for: Effect of chitosan and ε-Polylysine composite coating on postharvest quality maintenance and disease resistance of fresh Tremella fuciformis
Source: Food Chem X. 2026 Jun 3;37:104050. doi: 10.1016/j.fochx.2026.104050 (PMC13273120; doi:10.1016/j.fochx.2026.104050)
Supplement: Supplementary file 1 — Supplementary material [file mmc1.docx]

**Supplementary materials**

**Fig. S1.** Effects of CTS and ε-PL composite coating on the nutritional components of fresh *T. fuciformis*. Polysaccharide content (A); Vitamin C content (B); Dietary fiber content (C). Asterisks indicate significant differences compared to the control (***P* < 0.01).


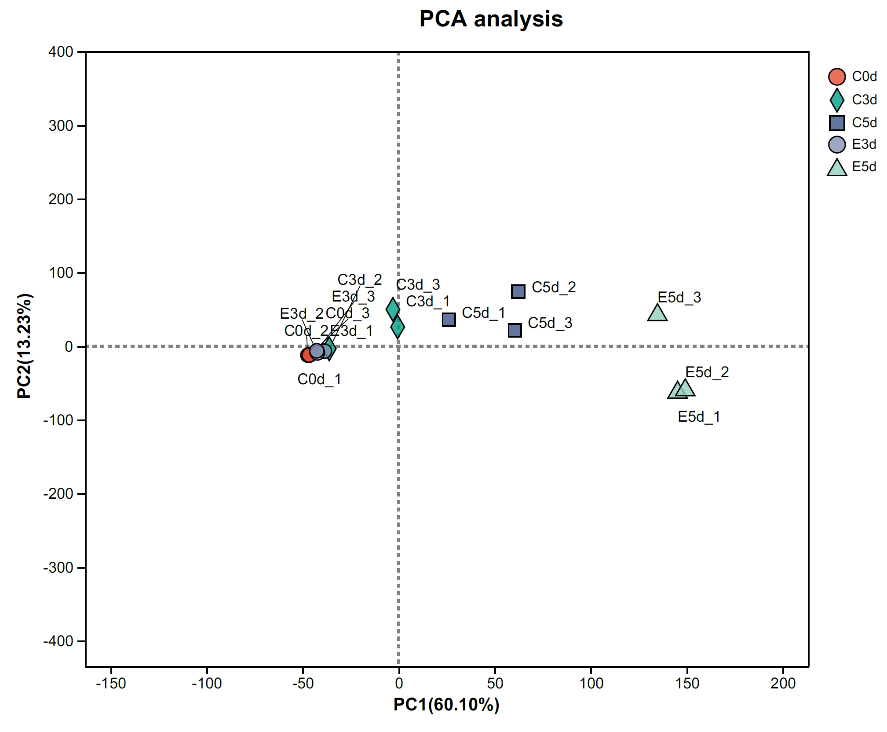


**Fig. S2.** Principal component analysis (PCA) of the transcriptomic data in fresh *T. fuciformis*. The plot demonstrates the clustering and high reproducibility of biological replicates from the control (C) and CTS + ε-PL treatment (E) groups during storage.


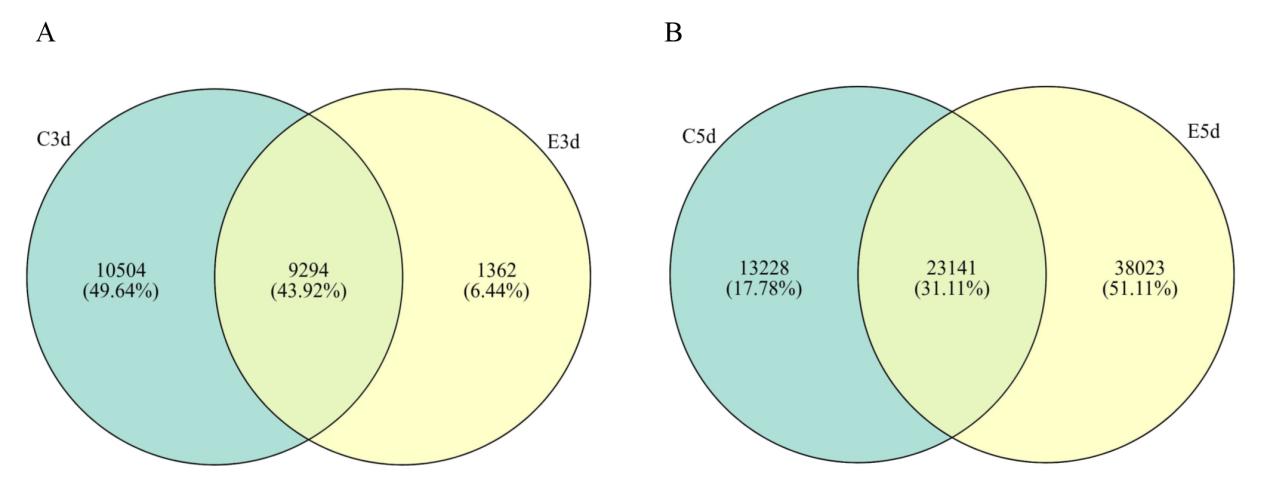


**Fig. S3.** Venn diagrams illustrating the distribution of differentially expressed genes (DEGs) in fresh *T. fuciformis*. The diagrams display the number of overlapping and unique DEGs between the control and CTS + ε-PL treatment groups on day 3 (A) and day 5 (B).


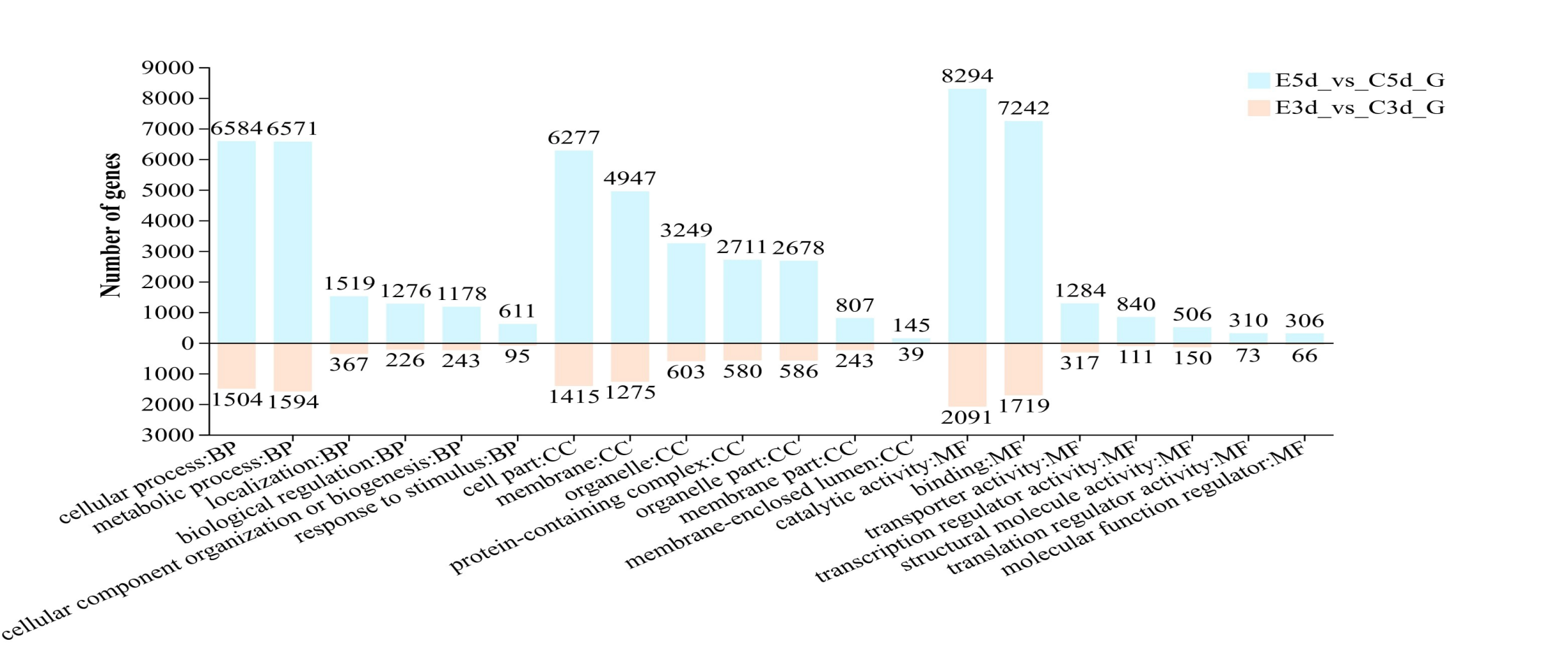


**Fig. S4.** Gene Ontology (GO) annotation and functional classification of the differentially expressed genes (DEGs) in fresh *T. fuciformis*. The bar chart displays the number of DEGs assigned to major functional categories across three main ontologies: Biological Process (BP), Cellular Component (CC), and Molecular Function (MF).
